# Predictive model for evolving density and viscosity gradients in band-forming ultracentrifugation

**DOI:** 10.1007/s00249-025-01759-7

**Published:** 2025-06-13

**Authors:** Lukas Dobler, Emre Brookes, Piotr Grodzki, Maciej Lisicki, Borries Demeler, Helmut Cölfen, Piotr Szymczak

**Affiliations:** 1 Department of Chemistry, Universität Konstanz, Konstanz, Germany; 2 Department of Chemistry and Biochemistry, University of Montana, Missoula, USA; 3 Institute of Theoretical Physics, Faculty of Physics, University of Warsaw, Warsaw, Poland; 4 Department of Chemistry and Biochemistry, University of Lethbridge, Lethbridge, Canada

**Keywords:** Analytical ultracentrifugation, Band-forming, Dynamic gradient, Solvent properties, Diffusion, Solvent mixing

## Abstract

Band-forming experiments allow the study of a wide variety of systems by overlaying two solutions with different densities in an analytical ultracentrifuge. Despite their potential benefits over other methods, these experiments are rarely used because all available fitting software encounters systematic errors, failing to account for the evolving gradient in density and viscosity due to diffusive mixing between the two layers. We develop and experimentally validate a predictive model for the purely diffusive mixing of two solutions in a cylindrical system. Capturing the space- and time-dependent evolution of density and viscosity in band-forming experiments, the model enhances their interpretation and underscores the need for analysis software to account for these dynamic changes.

**Supplementary Information:**

The online version contains supplementary material available at 10.1007/s00249-025-01759-7.

## Introduction

Analytical ultracentrifugation (AUC) is a first-principles experimental technique that is used to characterize nanoscale molecules in the solution state by recording their sedimentation profiles. It has been applied to a wide range of systems, including proteins (Edwards et al. 2020; Chaton and Herr 2015), viruses (Maruno et al. 2021; Wawra et al. 2023; Sternisha et al. 2023; Khasa et al. 2021), nucleic acids (Edwards et al. 2020; Ranasinghe et al. 2023; Urban et al. 2016) and polymers (Planken and Cölfen 2010; Diaz et al. 2015), as well as nanoparticles composed of metals (Völkle et al. 2015; Schneider and Cölfen 2019, 2020; Wawra et al. 2018; Thajudeen et al. 2017; Lopez et al. 2022; González-Rubio et al. 2021; Urban et al. 2016), semiconductors (Börger et al. 2000; Walter et al. 2017; Karabudak et al. 2016), insulators (Goertz et al. 2009; Mittal et al. 2010; Uttinger et al. 2020) or lipids (Zhao et al. 2024; Henrickson et al. 2021). Its versatility relies on the use of different detectors, with the two most popular types being multiwavelength (MWL) UV/Vis absorbance detectors (Karabudak et al. 2016; Pearson et al. 2018), and Rayleigh interference detectors (Schilling and Krause 2015). The MWL-detection allows one to study complex mixtures and distinguish species not only by sedimentation but also by their spectral properties, making it practical for biological and chemical samples (Maruno et al. 2021; Sternisha et al. 2023; Wawra et al. 2018; Lopez et al. 2022; Schneider and Cölfen 2019; Wawra et al. 2019; Henrickson et al. 2021; Karabudak et al. 2016; Völkle et al. 2015). In the past, various other detectors, such as Schlieren or fluorescence detectors, were commercially available and are still occasionally used (MacGregor et al. 2004; Wawra et al. 2019; Mächtle 1992, 1999; Xu and Cölfen 2021; Cölfen 2023). Although the most popular measurement mode is the traditional boundary experiment, often referred to as sedimentation velocity (SV) (Cölfen 2023), the capabilities of AUC are further extended by alternative experimental modes, such as speed ramp or density variation experiments.

An attractive alternative to traditional AUC techniques is the band-forming experiment (BFE). In contrast to a traditional setup, where the solute is initially uniformly distributed throughout the AUC cell at the beginning of the experiment, in a BFE the sample layer is placed on the top (closer to the axis of rotation) of the higher-density solution in the channel and subsequently centrifuged. BFE was first introduced by Vinograd et al. (1963) and requires the so-called Vinograd centerpieces, which are adapted by adding a reservoir near the top of the centerpiece channel. This reservoir is connected by a thin capillary to the solution channel. Upon commencement of centrifugation, the solution in the reservoir is transferred into the channel by centrifugal forces. To facilitate band formation, the solvent in the centerpiece channel must have a density higher than that of the solvent in the reservoir. Typically this density difference is created by (partially) replacing $\hbox {H}_{2}\hbox {O}$ in the channel solution with $\hbox {D}_{2}\hbox {O}$. This creates a stacking effect and leads to the formation of a sharp band (Vinograd et al. 1963; Vinograd and Bruner 1966a, b). A sharp Gaussian peak is then formed for a homogeneous sample; as it migrates away from the meniscus, it broadens as a result of diffusion, just like the sigmoidal boundary in a traditional SV experiment.

BFEs offer a convenient way to investigate particle mixtures, chemical reactions, or biological systems *in-situ*. Typically, the solution in the reservoir is up to ten times more concentrated but has a volume smaller by a factor of 40, resulting in less sample consumption compared to traditional sedimentation boundary experiments. A common application of BFE is active enzyme centrifugation, in which an enzyme solution is overlaid onto the substrate solution (Chou et al. 2011). The enzyme can be investigated in its active state without purification (Chou et al. 2011). For non-biological samples, BFE can freeze products of chemical reactions, for example, the early stages of nucleation of nanoparticles, by sedimentation away from the phase boundary. This allows one to physical separate different species or generations of particles. This separation capability is crucial for observing reaction progress and isolating specific phases without the interference of transient dynamics, that could affect sample integrity during experiments involving nanoparticles (Börger et al. 2000; Karabudak et al. 2016; Schneider and Cölfen 2019, 2020). By combining the advantages of BFE with powerful detectors such as UV/Vis-MWL absorption, subnanometer size resolution becomes possible, as demonstrated for metal (Völkle et al. 2015) and semiconductor (Karabudak et al. 2016) nanoparticles.

Schneider and Cölfen (2018) and Schneider et al. (2018) demonstrated that mixing the upper phase with the lower phase in BFE leads to the formation of gradients in both viscosity and density. While the latter is a critical necessary element in achieving stable band sedimentation (Vinograd et al. 1963; Vinograd and Bruner 1966a, b), it had been neglected in all prior analyses. Due to the differences in density and viscosity between light and heavy water, the apparent sedimentation and diffusion coefficients of an analyte change over time and spatial position until diffusion equilibrium is reached, with the concentrations of light and heavy water becoming uniform throughout the channel. While there are density gradient experiments in the AUC utilizing co-sedimenting additives, like $\hbox {CsCl}$ and Nycodenz, their analysis relies on data obtained when the system reaches equilibrium (Vinograd et al. 1963; Sternisha et al. 2023; Planken and Cölfen 2010). Furthermore, the experiment is only capable of determining the sample buoyant density, but not the sedimentation coefficient (Savelyev et al. 2023). In contrast, the BFE data is recorded when the system is out of equilibrium, with the gradient evolving over time due to $\hbox {D}_{2}\hbox {O}$ - $\hbox {H}_{2}\hbox {O}$ diffusion. Sedimentation transport can be neglected because the centrifugal force is not sufficient to sediment $\hbox {H}_{2}\hbox {O}$ molecules in $\hbox {D}_{2}\hbox {O}$ over the time span of the experiment. The time- and position-dependent changes of the solvent density and viscosity are not considered in common analysis software such as *sedfit* (Schuck 2000) and *UltraScan III* (Demeler and Gorbet 2016), which assume constant viscosity and density, and result in non-random and elevated residuals during data analysis. Recent studies utilizing BFE have simultaneously highlighted its advantages and limitations, leveraging its low sample consumption and high separational power to analyze adeno-associated virus vectors (Khasa et al. 2021; Maruno et al. 2023). The differences in sedimentation coefficients between SV-AUC and BFE-AUC reported by Maruno et al. highlight the challenges BFE data pose for standard analysis software. Gradient in viscosity and density can lead to artificial peak broadening, which may be misinterpreted as additional species.

While the problem of dynamic gradients was identified earlier (Schneider and Cölfen 2018; Schneider et al. 2018), no model has been proposed to accurately simulate the changes in density and viscosity. This lack of a theoretical description has hindered further investigation into how BFEs are influenced by these evolving gradients.

In this paper, we present a mathematical model for the evolution of dynamic gradients in density and viscosity to aid in the analysis of BFE data using Lamm equation modeling. The theoretical framework is based on the diffusion-driven mixing of light and heavy water. We validate the model predictions with recorded experimental data and numerical calculations to highlight the pronounced effect of cell geometry on viscosity and density gradients.

## Experimental section

### Materials

$\hbox {H}_{2}\hbox {O}$ was sourced from a Milli-Q Synthesis A10 system, which was equipped with a Quantum EX Ultrapure Organex cartridge (Millipore). Deuterium oxide ($\hbox {D}_{2}\hbox {O}$) was obtained from Sigma Aldrich and used without any further purification steps.

### Data acquisition

The sample, $\hbox {H}_{2}\hbox {O}$, was measured in a charcoal-filled Epon band-forming centerpiece with a $12~\text {mm}$ path length, purchased from Beckman. This has a small reservoir that holds up to a volume of $15~\upmu \text {L}$. The reservoir is connected to the sample channel through a narrow capillary. The centerpiece was scanned and a channel angle of $2.5^{\circ }$ was determined.

The experiment was performed by overlaying $\hbox {H}_{2}\hbox {O}$ over $\hbox {D}_{2}\hbox {O}$ during acceleration to 3000 rpm. The analytical ultracentrifuge was started after 1 h of equilibration at $20^{~\circ }\text {C}$, with an acceleration rate of 600 rpm per minute. Data acquisition was carried out using an advanced Rayleigh interference optics (AIDA) developed by Nanolytics (Schilling and Krause 2015). In addition, the instrument was modified to allow data acquisition during the acceleration phase to directly observe the overlay process (Schneider et al. 2018). Differences between the custom-built analytical ultracentrifuge used in this study and standard commercial instruments have been documented in the literature (Bhattacharyya et al. 2006; Strauss et al. 2008; Walter et al. 2014; Schilling and Krause 2015; Walter and Peukert 2016; Pearson et al. 2018; Schneider and Cölfen 2018; Schneider et al. 2018).

### Mathematical model

**Fig. 1:**
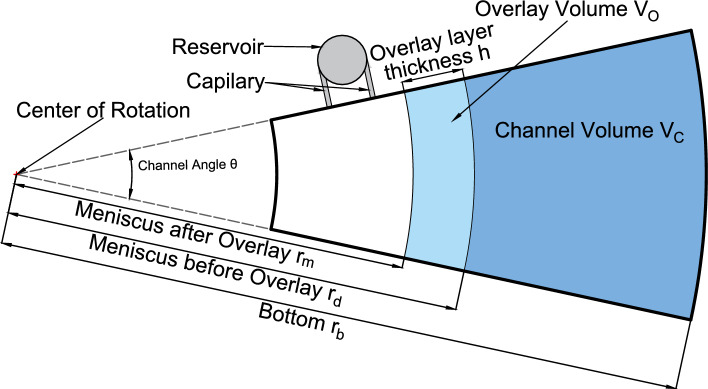
Schematic drawing of a band-forming centerpiece channel with reservoir

To describe the concentration gradient in the cell, we consider a section of an annulus with meniscus radius $r_m$ (after the overlay) and bottom of the channel with the radius $r_b$. A schematic drawing of a channel can be found in Fig. 1. Assuming axial symmetry, the diffusion equation for the concentration *c* of the solvent is written in the radial coordinate *r* as

1 $$\begin{aligned} \frac{\partial ^2 c}{\partial r^2} + \frac{1}{r} \frac{\partial c}{\partial r} = \frac{1}{D} \frac{\partial c}{\partial t}, \end{aligned}$$

supplemented by the reflecting (zero current) boundary conditions

2 $$\begin{aligned} \frac{\partial c}{\partial r} = 0 \qquad \text {at} \ \ r=\{r_m,r_b\}, \end{aligned}$$

and an initial condition after a disturbance free overlay

3 $$\begin{aligned} c(r,t=0) = F(r) = {\left\{ \begin{array}{ll} c_i & \ \ \ r_m \le r\le r_m+h\\ 0 & \ \ \ r_m+h< r < r_b \end{array}\right. }, \end{aligned}$$

with *h* being the thickness of the upper solvent layer and $c_i$ being the concentration of the less dense solvent in the reservoir, in this case $\hbox {H}_{2}\hbox {O}$. The thickness *h* can be calculated based on the overlay volume and the geometric characteristics of the centerpiece. We solve Eq. (1) using an eigenfunction expansion

4 $$\begin{aligned} c(r, t) = \sum _{n} a_n R(\beta _n,r) \text {exp}\left( -D \beta _n^2 t\right) , \end{aligned}$$

where $R(\beta _n,r)$ is the solution of

5 $$\begin{aligned} \frac{\partial ^2 R}{\partial r^2} + \frac{1}{r} \frac{\partial R}{\partial r} + \beta _n^2 R = 0, \end{aligned}$$

which is the Bessel equation of zero order. The general solution of Eq. (5) is

6 $$\begin{aligned} R(\beta _n, r) = A_n J_0(\beta _n r)+B_n Y_0(\beta _n r), \end{aligned}$$

where $J_\nu$ and $Y_\nu$ denote the Bessel functions of the first and second kind with $\nu$ specifying the order (Hahn and Özişik 2012).

Imposing the boundary conditions on Eq. (6) at $r_m$ and $r_b$ leads to $A_n = Y_1 (\beta _n r_b)$, $B_n = J_1 (\beta _n r_b)$, and the following transcendental equation for $\beta _n$

7 $$\begin{aligned} J_1\left( \beta _n r_m\right) Y_1\left( \beta _n r_b\right) - J_1\left( \beta _n r_b\right) Y_1\left( \beta _n r_m\right) =0. \end{aligned}$$

Finally, the expansion coefficients $a_n$ can be found by expressing the initial condition, *F*(*r*), in terms of the eigenfunctions $R(\beta _n, r)$. This leads to the following solution of the original problem

8 $$\begin{aligned} c(r,t)= & c_\text {eq} + \sum _{n} \frac{1}{N\left( \beta _n\right) } R\left( \beta _n, r\right) \text {exp}\left( -D \beta _n^2 t\right) \nonumber \\ & \int _{r_m}^{r_b} R\left( \beta _n, r'\right) F\left( r'\right) r' {\rm d}r' \end{aligned}$$

with the norm $N(\beta _n)$ given by

9 $$\begin{aligned} \frac{1}{N(\beta _n)}=\frac{\pi ^2}{2} \frac{\beta _n^2 J^{\prime 2}_0\left( \beta r_m\right) }{J^{\prime 2}_0\left( \beta _n r_m\right) -J^{\prime 2}_0\left( \beta r_b\right) }. \end{aligned}$$

The first summand in (8), $c_\text {eq}$, is a constant term related to the first eigenvalue $\beta _0=0$. Physically, it corresponds to the equilibrium, uniform concentration of $\hbox {H}_{2}\hbox {O}$ in the system, which can be calculated from the volumes loaded into the cell channel and reservoir.

With the initial condition given by Eq. (3) the integral in Eq. (8) takes the form

10 $$\begin{aligned} \begin{aligned}&\int _{r_m}^{r_b} R\left( \beta , r'\right) F\left( r'\right) r' \textrm{d}r' = \\&\quad \left\{ Y_1 \left( \beta r_b\right) \left[ r_d J_1 \left( \beta r_d\right) -r_m J_1 \left( \beta r_m\right) \right] \right. \\&\quad \left. + J_1 \left( \beta r_b\right) \left[ r_m Y_1 \left( \beta r_m\right) - r_d Y_1 \left( \beta r_d\right) \right] \right\} \frac{c_i}{\beta }. \end{aligned} \end{aligned}$$

where $$r_d=r_m +h$$.

The calculation of the concentration profiles was carried out using the approach described above, with a C++ code developed by us to facilitate future integration into existing analysis software. The GNU scientific library was used to implement the Bessel functions. The eigenvalues were determined using a secant solver on the interval from 0.01 to 5000 and all found eigenvalues were used for the calculations.

For the diffusion of $\hbox {H}_{2}\hbox {O}$ and $\hbox {D}_{2}\hbox {O}$, we assumed the mutual diffusion coefficient to be $D = 1.8 \times 10^{-5}\ \text {cm}^2/{\text {s}}$ as described in the Supporting Information (SI). The density and viscosity of the water and heavy water were taken from the parametrizations published in reference (Philo 2023). The channel was modeled as a section of a hollow cylinder with a height of 12 mm and an outer diameter of 7.166 cm and an inner diameter of 6.163 cm.

### Thickness of the overlay layer

When the content of the reservoir is layered on top of the solution in the channel, the meniscus rises slightly to the position $r_m$. The thickness of this thin layer is a key parameter for the calculation of the concentration profiles of $\hbox {H}_{2}\hbox {O}$ during the experiment. Previous studies considered only the effect of different overlay volumes on the thickness, without accounting for the channel angle or the volume within the channel $V_C$ (Schneider et al. 2018). To determine the layer thickness, users had to follow the experimental protocol outlined by Schneider et al. (2018) This protocol required recording scans during the acceleration phase, which was not feasible without modified hardware and software.

Alternatively, the overlay thickness, *h*, can be calculated from a simple geometrical consideration, using the experimentally observed meniscus position, $r_m$, the channel angle (in radians), $\theta$, channel height, *l*, and the overlay volume, $V_O$, as

11 $$\begin{aligned} h(\theta ,V_O,r_m) = \sqrt{r_m^2 + \frac{2}{\theta l} V_O }-r_m. \end{aligned}$$

With the knowledge of the volume filled into the column $V_C$, the sector angle $\theta$ and overlaid volume $V_O$, the meniscus shift *h* can also be calculated as

12 $$\begin{aligned} h(r_b, \theta , V_C, V_O) = \sqrt{r_b^2- \frac{2}{\theta l}V_C} - \sqrt{r_b^2 - \frac{2}{\theta l} \left( V_C + V_O\right) }. \end{aligned}$$

It is important to note that $V_C$ and $V_O$ are the planned volumes —i.e., the intended amounts to be filled. However, due to practical uncertainties in experimental preparation (e.g., incomplete emptying of reservoirs, residual liquid in pipettes, or acceleration-related deviations), these volumes may not reflect the actual values. As these errors are not reliably quantifiable, calculations based on these volumes involve an unknown uncertainty. The benefit of Eq. (11) compared to Eq. (12) is that the uncertainty of the channel volume $V_C$ is avoided in favor of the experimentally determined meniscus position. In addition, the meniscus position is already needed for the analysis of the AUC data. While the combination of both equations allows the calculation of the overlay thickness and the channel angle, measuring the channel angle in the centerpiece provides more precise results. This further reduces the uncertainty of the calculated overlay thickness.

## Results and discussion

### Calculation of concentration gradients

**Fig. 2:**
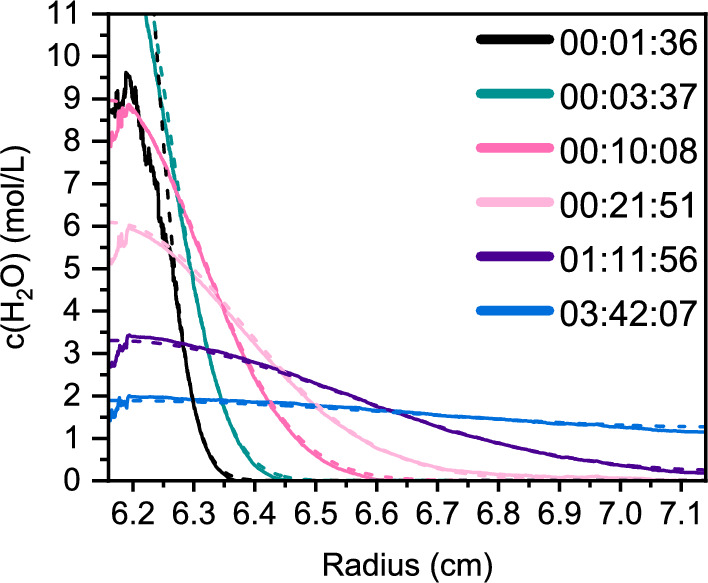
The experimental interference (solid) and theoretical (dashed) profiles at different time points for the overlay of 10 $\upmu \text {L}$ $\hbox {H}_{2}\hbox {O}$ onto $\hbox {D}_{2}\hbox {O}$

We compare the analytical predictions of the concentration profiles based on Eq. (8) with the recorded Rayleigh-interference data for a 10 $\upmu \text {L}$ overlay volume. The comparison is presented in Figure 2.

The comparison between the analytical calculations and the measured experimental profiles shows good agreement at later times (teal, bright pink, light pink, dark purple, blue), but shows a deviation at early times. This is because the rate of acceleration during the initial stage is much lower than in typical AUC experiments. Consequently, the timing of the overlay is primarily speed-dependent (Schneider and Cölfen 2018; Schneider et al. 2018). The presented model assumes the overlay to occur instantaneously, at $t = 0$. However, in the experimental data, it took three minutes to reach the final speed. The speed profile of this experiment is provided in the supporting information, Fig. S1. This slow acceleration explains the deviation observed, particularly in the earliest scan (black line in Fig. 2). However, the high accuracy of prediction at later times validates the accuracy of the $t = 0$ overlay assumption for experiments with low acceleration.

**Fig. 3:**
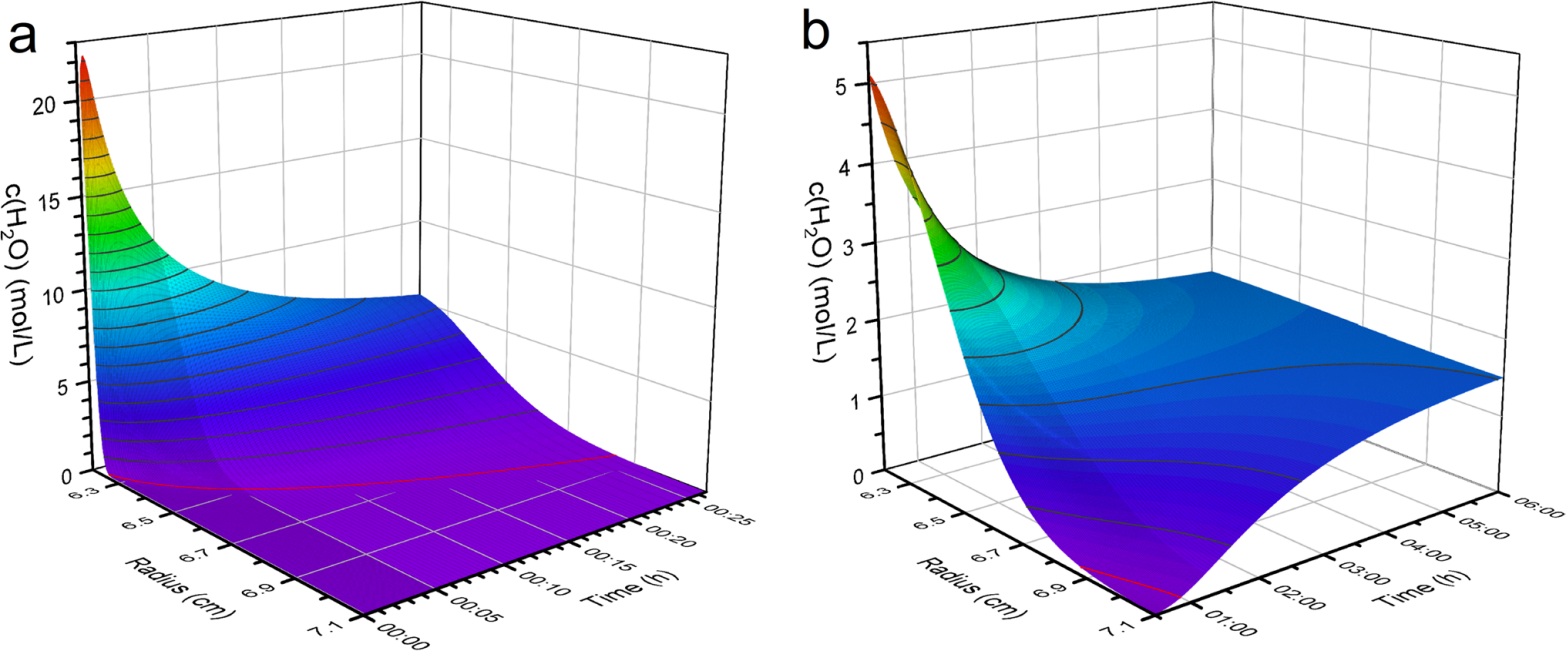
Concentration profiles after the overlay of $10~\upmu \text {L}$ $\hbox {H}_{2}\hbox {O}$ onto $\hbox {D}_{2}\hbox {O}$. The profiles are shown shortly after the overlay (**a**) and at longer times (**b**). The red line marks the concentration of $0.1~{\text {mol}}/{\text {L}}$ $\hbox {H}_{2}\hbox {O}$

Additionally, the observed meniscus is broader due to the low rotational speed and the associated lower centrifugal forces, which counteract surface tension to a lesser extent. This results in a stronger surface curvature, leading to a less defined meniscus. Consequently, this causes a broader concentration profile of $\hbox {H}_{2}\hbox {O}$ at lower rotation speeds compared to the same setups at higher rotation speeds. This affects especially scans taken during acceleration compared to scans taken after the end of the acceleration phase. This contributes in Fig. 2 to the missing area between the simulation profile and the measured signal for the earliest scan (black). The broadening of the meniscus dependent on the rotational speed was previously observed by Schneider et al. (2018) Earlier attempts to model the concentration profiles struggled to accurately represent the geometry of the system (Schneider and Cölfen 2018). The method described here correctly accounts for the shape of the centerpiece, ensuring a precise description of the radial dilution effect observed in AUC experiments. The radial dilution results in a steeper concentration profile compared to models assuming rectangular geometry, as manifested by an extra drift term, $({D}/{r})({\partial c}/{\partial r})$ in the diffusion equation, Eq. (1), which is not present in its rectangular counterpart. The evolution of the concentration gradient over time can be calculated, as shown in Fig. 3. The concentration of $\hbox {H}_{2}\hbox {O}$ after 6 h is still slightly higher at the meniscus compared to the bottom. This emphasizes the importance of accounting for the effect of the dynamic gradient on the solvent of a BFE.

Given the concentration of $\hbox {H}_{2}\hbox {O}$, the density or viscosity of the $\hbox {H}_{2}\hbox {O}$–$\hbox {D}_{2}\hbox {O}$ mixture can be calculated (Philo 2023). The general equation can be simplified for isotopic mixtures of water yielding a volume-fraction-weighted average for both density and viscosity (Steckel and Szapiro 1963; Philo 2023). By rewriting the volume fractions in terms of concentrations, the density can be expressed as

13 $$\begin{aligned} \rho (c_{\hbox {H}_{2}\hbox {O}}) = \rho _{\hbox {D}_{2}\hbox {O}} + \frac{\rho _{\hbox {H}_{2}\hbox {O}}-\rho _{\hbox {D}_{2}\hbox {O}}}{c_{self,\hbox {H}_{2}\hbox {O}}}c_{\hbox {H}_{2}\hbox {O}} \end{aligned}$$

and the viscosity as

14 $$\begin{aligned} \eta (c_{\hbox {H}_{2}\hbox {O}}) = \eta _{\hbox {D}_{2}\hbox {O}} + \frac{\eta _{\hbox {H}_{2}\hbox {O}}-\eta _{\hbox {D}_{2}\hbox {O}}}{c_{self,\hbox {H}_{2}\hbox {O}}}c_{\hbox {H}_{2}\hbox {O}}, \end{aligned}$$

where $$c_{self,\hbox {H}_{2}\hbox {O}} = \rho _{\hbox {H}_{2}\hbox {O}}/M_{\hbox {H}_{2}\hbox {O}} = 55.56~\text {mol}/\text {L}$$.

### Impact of the channel angle

The channel angle plays a crucial role in several aspects of the analysis. It directly influences the radial dilution, introducing the dependence of volumes on radial positions. Additionally, the channel angle determines the thickness of the overlay layer, an essential parameter for calculating concentration profiles in Eq. (11).

**Fig. 4:**
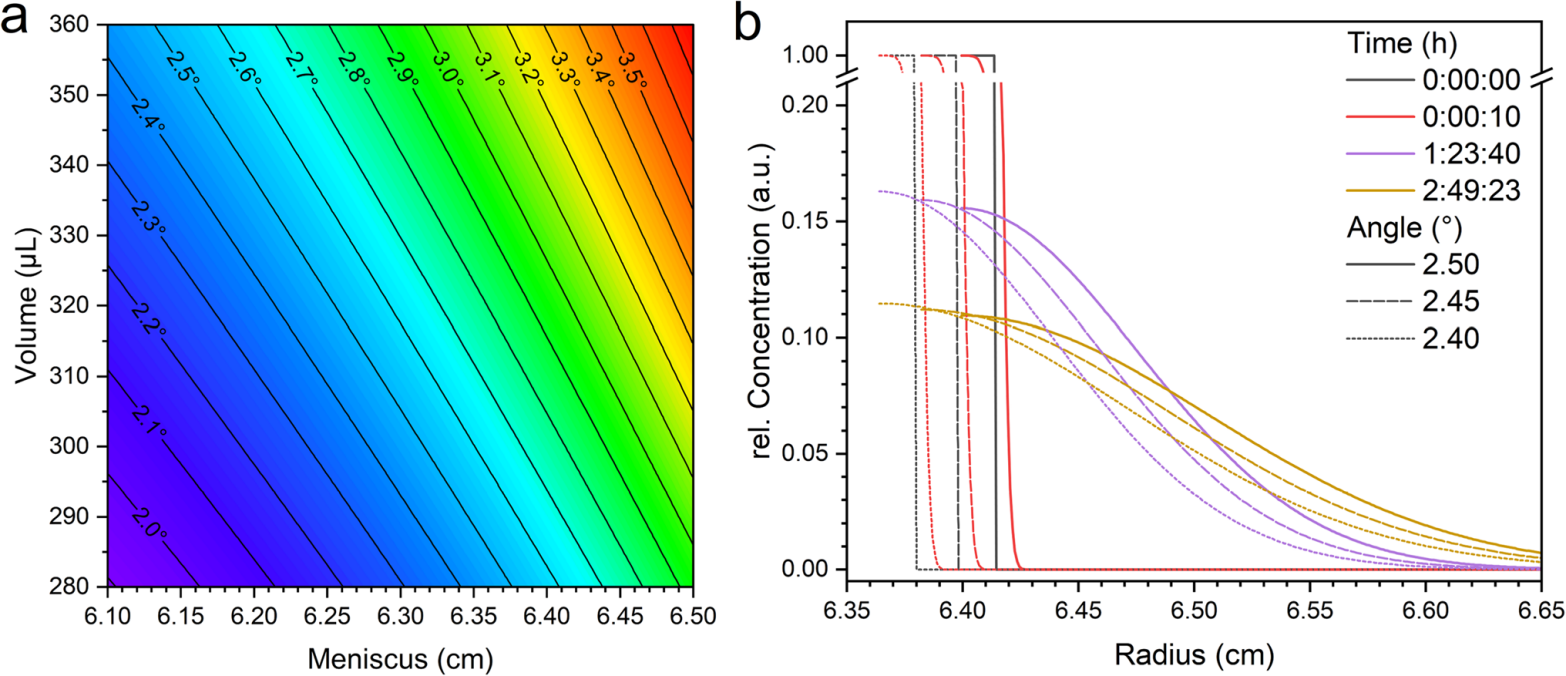
Calculation of the channel angle (**a**) as a function of the volume and the meniscus position after overlay in a channel with a bottom of $7.166~\text {cm}$. Comparison of the concentration profiles (**b**) for the same volume after overlay (const $V_C+V_O$) for $2.5^{\circ }$ (solid), $2.45^{\circ }$ (dashed) and $2.4^{\circ }$ (dotted)

The channel angle can be calculated based on the bottom radius, the meniscus position after the overlay, and the total volume, with the results shown in Fig. 4a. However, calculating the angle of the channel from experimental data requires more precise knowledge of the radial position of the meniscus and the volume than is currently achievable. The volume can deviate from the theoretical value due to the small portion of the overlaying solution that might remain in the reservoir or due to loading errors, which are hard to quantify in general. Typically, the uncertainty for the radial positions due to the optics is around $10~\upmu \text {m}$ (Clodfelter and Schwartz 2017). However, the actual measurement error for the position of the meniscus is even greater, due to factors such as the curvature of the meniscus. Note that a meniscus shift of $50~\upmu \text {m}$ results in a change in the channel angle of around $0.01^{\circ }$. Such a difference can significantly affect the concentration profiles when modeling the system, as demonstrated in Fig. 4. Although the volume after the overlay ($V_C+V_O$) remains constant across the different cases shown in the figure, the position of the meniscus shifts due to the variations in the channel angle. Fig. 4 demonstrates the importance of obtaining a reliable value for the channel angle to ensure precise calculations. The best way to achieve this is through a direct measurement of the channel angle, which is sufficient for accurate predictions, as shown in Fig. 2, and is preferred over back-calculating the angle of the channel from the radial position of the meniscus and the overlay volume. The importance of measuring the channel angle is further underlined by the fact that centerpieces from different manufacturers can vary by as much as 1 degree in their channel angles. Failure to account for these variations when modeling individual channels would introduce significant errors.

## Conclusion

In this study, we developed a mathematical model capable of predicting the diffusive mixing of two solution layers in a system with cylindrical geometry. This model closely matches experimental data of the overlay of $\hbox {H}_{2}\hbox {O}$ onto $\hbox {D}_{2}\hbox {O}$, enabling, for the first time, an accurate description of solution density and viscosity throughout the entire experiment.

The key parameters in this model are the channel angle and the thickness of the overlay layer, both of which significantly influence the accuracy of the concentration profiles, as demonstrated by calculations for different channel angles. We also explored different approaches for estimating these parameters. Determining the overlay thickness or channel angle based solely on the position of the channel bottom and meniscus, along with the channel volume, was found to be insufficiently precise. Instead, we recommend a direct measurement of the channel angle before the experiment. This method eliminates the need for specialized hardware or software, making it accessible to researchers using standard laboratory equipment while ensuring accurate results.

The presented description for dynamic gradients, based on the diffusive mixing of two phases, is a significant step towards enabling researchers to analyze BFE. With this model, for the first time since the development of BFE 60 years ago, the dynamic gradients in density and viscosity can be accurately predicted. The model is also applicable to other solvents, provided that the diffusion coefficient, density, and viscosity of the solvent mixture are known.

Looking ahead, the current fitting algorithms in AUC analysis software will need to be adapted to accommodate position- and time-dependent variations in density and viscosity. With this model implemented, the analysis software should be capable of analyzing BFEs. Given the demonstrated accuracy for the prediction of gradients, it is plausible that Rayleigh interference detectors could be corrected for the signal caused by the dynamic gradient in the recorded data. This opens the opportunity to use Rayleigh interference detectors for BFEs of samples that are not detectable with UV/Visible detectors

## Supplementary Information

Below is the link to the electronic supplementary material.

Supplementary file 1 (pdf 329 KB)

## Data Availability

All figure data and the raw interference data from the AUC experiment are available on Zenodo (10.5281/zenodo.15601163).
