# Supplementary material for: Predictive model for evolving density and viscosity gradients in band-forming ultracentrifugation
Source: Eur Biophys J. 2025 Jun 13;54(6):295–303. doi: 10.1007/s00249-025-01759-7 (PMC12552396; doi:10.1007/s00249-025-01759-7)
Supplement: Supplementary file 1 — (pdf 329 KB) [file 249_2025_1759_MOESM1_ESM.pdf]

Supporting Information: Predictive model for evolving density and viscosity gradients in band-forming ultracentrifugation

Lukas Dobler,[†] Emre Brookes,[‡] Piotr Grodzki,[¶] Maciej Lisicki,[¶] Borries Demeler,[§,‡] Helmut Cölfen,[†,||] and Piotr Szymczak[*,¶]

†*Universität Konstanz, Universitätsstraße 10, 78457 Konstanz, Germany*

‡*Department of Chemistry and Biochemistry, University of Montana, Missoula, Montana, USA*

¶*Institute of Theoretical Physics, Faculty of Physics, University of Warsaw, Warsaw, Poland*

§*Department of Chemistry and Biochemistry, University of Lethbridge, Lethbridge, AB, T1K3M4, Canada*

||*Deceased*

E-mail: piotrek@fuw.edu.pl

Contents

1 Speed profile of overlay experiment **3**

2 Determination of the mutual diffusion coefficient of H_2O and D_2O **3**

1 Speed profile of overlay experiment

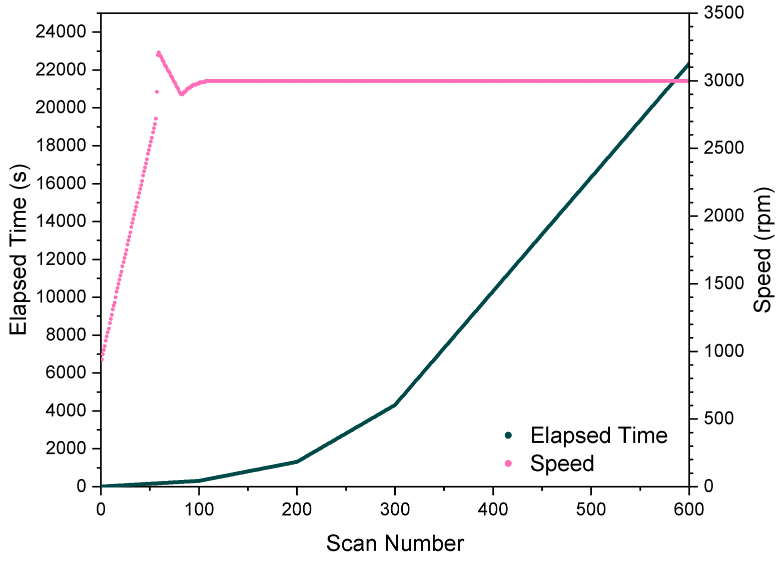

Figure 1: The time (green) and speed (pink) of the measured scans for the overlay of 10 μL H_2O onto D_2O.

2 Determination of the mutual diffusion coefficient of H_2O and D_2O

The self-diffusion coefficients for H_2O and D_2O were obtained by averaging over numerous literature values, which can be found in Table 1. The values were plotted as $ln(D)$ against T^{-1} and fitted with a linear function to obtain the diffusion coefficient as a function of temperature. $D_{\mathrm{H_2O}}(T) = 2070 \pm 28 \times T^{-1} + 12.96 \pm 0.09$ was obtained for H_2O with an adjusted R-Square of 0.9929 and $D_{\mathrm{D_2O}}(T) = 2221 \pm 113 \times T^{-1} + 12.68 \pm 0.37$ for D_2O with an adjusted R-Square of 0.9822. The self-diffusion coefficient under experimental conditions of 20 °C was therefore assumed as 2.01×10^{-9} m^2/s for H_2O and 1.59×10^{-9} m^2/s for D_2O. The mutual diffusion coefficient of H_2O and D_2O was estimated as the average of both self-diffusion coefficients 1.80×10^{-9} m^2/s.

Table 1: Used self-diffusion coefficients of H_2O and D_2O from literature

Temperature [K]	$D_{D_2O}[m^2/s]$	$D_{H_2O}[m^2/s]$	Source
273.15		1.130E-9	[1]
274		1.149E-9	[2]
277		1.276E-9	[2]
278		1.281E-9	[3]
278		1.426E-9	[4]
278	1.015E-9	1.313E-9	[2]
283		1.675E-9	[4]
283.15		1.536E-9	[1]
288		1.970E-9	[4]
288		1.777E-9	[2]
288.15		1.766E-9	[5]
293.15		2.022E-9	[1]
293.15		2.025E-9	[5]
295	1.690E-9	2.140E-9	[6]
298		2.248E-9	[3]
298		2.570E-9	[4]
298	1.872E-9	2.229E-9	[2]
298.15		2.296E-9	[1]
298.15		2.299E-9	[5]
300	1.930E-9	2.360E-9	[6]
303.15		2.590E-9	[1]
303.15		2.597E-9	[5]
305	2.180E-9	2.640E-9	[6]
308		2.919E-9	[2]
308.15		2.895E-9	[5]
310	2.400E-9	2.840E-9	[6]
313.15		3.240E-9	[1]
313.15		3.222E-9	[5]
318	2.650E-9	3.290E-9	[6]
318		3.491E-9	[3]
318	2.979E-9	3.575E-9	[2]
318.15		3.601E-9	[5]
323.15		3.968E-9	[1]
323.15		3.983E-9	[5]
329.15		4.444E-9	[5]
333.15		4.772E-9	[1]
343.15		5.646E-9	[1]
353.15		6.582E-9	[1]
363.15		7.578E-9	[1]
373.15		8.623E-9	[1]

References

(1) Easteal, A. J.; Price, W. E.; Woolf, L. A. Diaphragm cell for high-temperature diffusion measurements. Tracer Diffusion coefficients for water to 363 K. *Journal of the Chemical Society, Faraday Transactions 1: Physical Chemistry in Condensed Phases* **1989**, *85*, 1091–1097.

(2) Mills, R. Self-diffusion in normal and heavy water in the range 1-45°. *Journal of Physical Chemistry* **1973**, *77*, 685–688.

(3) Meng, W.; Xia, Y.; Chen, Y.; Pu, X. Measuring the mutual diffusion coefficient of heavy water in normal water using a double liquid-core cylindrical lens. *Scientific Reports 2018 8:1* **2018**, *8*, 1–7.

(4) Wang, J. H. Self-diffusion coefficients of water. *Journal of Physical Chemistry* **1965**, *69*, 4412.

(5) Holz, M.; Heil, S. R.; Sacco, A. Temperature-dependent self-diffusion coefficients of water and six selected molecular liquids for calibration in accurate 1H NMR PFG measurements. *Physical Chemistry Chemical Physics* **2000**, *2*, 4740–4742.

(6) Dahal, U.; Adhikari, N. P. Molecular dynamics study of diffusion of heavy water in normal water at different temperatures. *Journal of Molecular Liquids* **2012**, *167*, 34–39.
